# Ellagic Acid Suppresses ApoB Secretion and Enhances ApoA-1 Secretion from Human Hepatoma Cells, HepG2

**DOI:** 10.3390/molecules26133885

**Published:** 2021-06-25

**Authors:** Ayana Ieda, Maki Wada, Yuuki Moriyasu, Yuuko Okuno, Nobuhiro Zaima, Tatsuya Moriyama

**Affiliations:** 1 Department of Applied Biological Chemistry, Graduate School of Agriculture, Kindai University, 3327-204, Naka-machi, Nara 631-8505, Japan; ayana227775@gmail.com (A.I.); wdkdgradsch@gmail.com (M.W.); mrmr874g@gmail.com (Y.M.); moriyama65@gmail.com (Y.O.); zaima@nara.kindai.ac.jp (N.Z.); 2 Agricultural Technology and Innovation Research Institute, Kindai University, 3327-204, Naka-machi, Nara 631-8505, Japan

**Keywords:** ellagic acid, apoB, VLDL, apoA-1, HDL, HepG2, MTP, hepatoma, secretion

## Abstract

The effect of ellagic acid (EA), a naturally occurring polyphenolic compound, on the secretion of apolipoproteins from human hepatocytes, HepG2, was investigated. The levels of apoB and apoA-1 secreted in the cell culture medium were determined by sandwich ELISA. EA did not affect cell viability at the tested concentrations (up to 50 µM). EA suppressed the secretion of apoB and enhanced that of apoA-1 from HepG2 cells. However, cellular apoB levels were increased, suggesting that EA inhibited the trafficking of apoB during the process of secretion. In contrast, the increase in the cellular levels of apoA-1 was consistent with its secreted levels. These results indicate that EA inhibits the secretion of apoB from hepatocytes and increases the secretion of apoA-1. Both of these effects are beneficial for lipoprotein metabolism in the prevention of lifestyle-related diseases. The detailed mechanism underlying these effects of EA on lipoprotein metabolism should be elucidated in the future, but this naturally occurring polyphenolic compound might be antihyperlipidemic. Based on these results, EA is suggested as a candidate food-derived compound for the prevention of hyperlipidemia.

## 1. Introduction

Lipids play various roles in maintaining the physical functions of the body and are essential for vital activities. Triglycerides (TGs) act as storage forms of energy, and cholesterol is a component of the plasma membrane and a raw material in the synthesis of steroid hormones. However, excessive intake of such lipid components leads to diseases, such as obesity and hyperlipidemia, fatty liver disease, and arteriosclerosis.

Lipoproteins are primarily responsible for the transport of lipids in the blood. This lipid–protein complex is comprised of a shell of apolipoproteins, phospholipids, and cholesterol around TGs and cholesterol esters. According to their specific gravity, lipoproteins are classified into chylomicrons, very low-density lipoproteins (VLDLs), low-density lipoproteins (LDLs), and high-density lipoproteins (HDLs). The major apolipoprotein of VLDLs and LDLs is apolipoprotein B (apoB) and the major apolipoprotein HDLs is apolipoprotein A-I (apoA-1), which is synthesized in the liver and small intestine [1].

VLDLs and LDLs are responsible for supplying TGs and cholesterol, which are biosynthesized in the liver, to peripheral tissues. Conversely, HDLs are responsible for the reverse transport of cholesterol from peripheral tissues to the liver. Thus, LDLs and HDLs have opposing effects on the accumulation of cholesterol in the blood vessels. Elevated LDL cholesterol and low HDL cholesterol levels are major risk factors for the development and progression of atherosclerosis and coronary heart disease [2]. These two apolipoproteins (apoB and apoA-1) secreted by the liver play an important role in lipid homeostasis in vivo. Therefore, dietary factors that suppress the secretion of VLDLs, including apoB, and enhance the secretion of apoA-1 may be candidates for the prevention of diseases resulting from dyslipidemia. As of date, several dietary components regulating the secretion of VLDLs (apoB) from hepatocytes have been reported. For example, components of polyphenol-rich red wine significantly reduce the secretion of VLDLs from hepatocytes [3].

**Figure 1:**
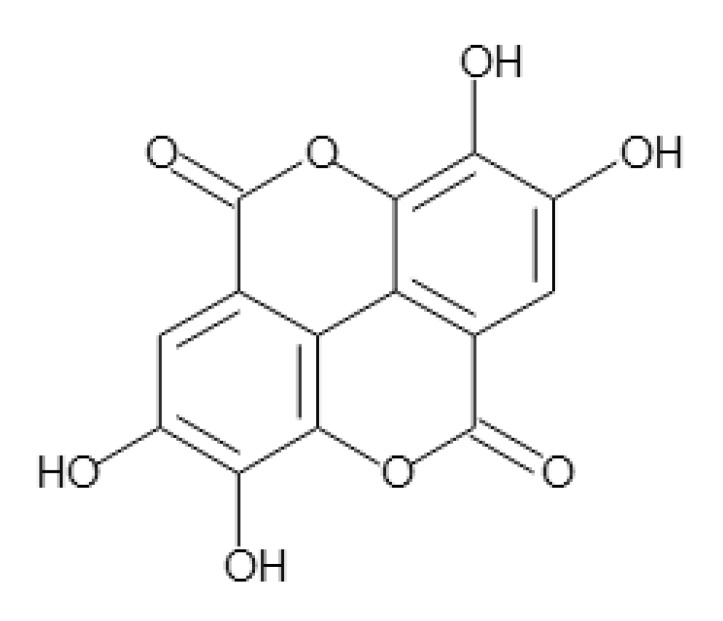
Chemical structure of ellagic acid.

Pomegranate (*Punica granatum*, Punnicaceae) contains a variety of phytochemicals, including polyphenols, anthocyanins, and tannins [4,5]. The consumption of pomegranate by patients with type 2 diabetes has been reported to reduce their levels of lipid peroxides and serum cholesterol and to protect against cardiovascular disease [6,7]. We also reported that the ellagic acid (EA; Figure 1) present in pomegranate suppresses the secretion of resistin from the adipocytes [8]. We showed that EA might interfere with the secretory processes without affecting the expression of resistin mRNA and might inhibit the secretion of resistin. In this context, in the present study, we investigated whether EA also affects the secretion of lipoproteins (apoB and apoA-1) from the hepatocytes using a human hepatoma cell line, HepG2.

## 2. Results

### 2.1. Effect of EA on the Viability of HepG2 Cells

**Figure 2:**
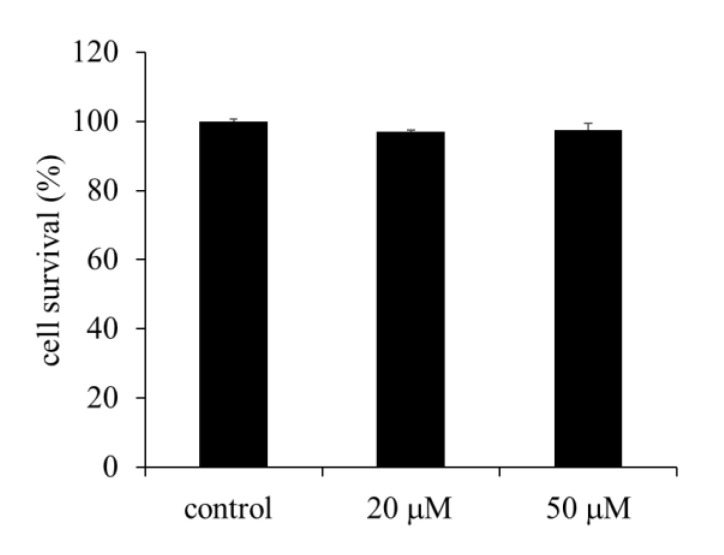
Effect of ellagic acid (EA) on the survival of HepG2 cells. The cells were exposed to EA at the indicated concentrations and cell survival was determined using CellTiter 96 AQ_ueous_ One^TM^ Solution after 24 h. Values are expressed as mean ± SE (*n* = 3).

The viability of HepG2 cells incubated with two different concentrations of EA was tested using CellTiter 96 AQ_ueous_ One^TM^ Solution (Promega Corporation, Fitchburg, WI, USA). No inhibition of cell growth was observed up to an EA concentration of 50 µM (Figure 2).

### 2.2. Effect of EA on the Secretion of VLDL (apoB) from HepG2 Cells

**Figure 3:**
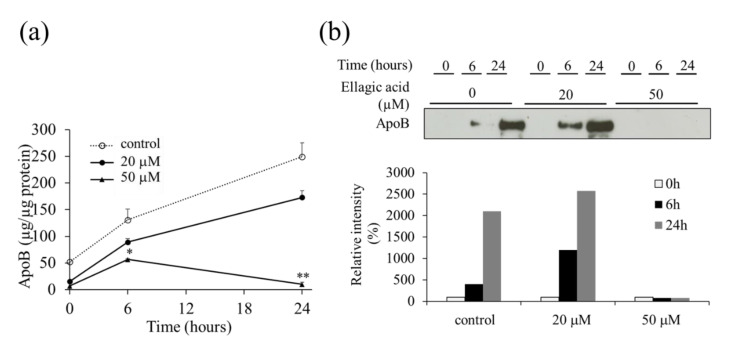
Effect of ellagic acid (EA) on the secretion of the very low-density lipoprotein (apoB) from HepG2 cells treated for 24 h. (**a**) Concentration of apoB secreted from HepG2 cells treated with EA in the culture medium was determined by ELISA as described in “Materials and Methods”. (**b**) Western blot analysis showing the levels of apoB secreted from HepG2 cells treated with EA in the culture medium. Values are expressed as mean ± SE (*n* = 3). * *p* < 0.05, ** *p* < 0.01 vs. control group (without EA treatment). The data was expressed by comparing band intensities in each concentration of ellagic acid to the concentration of relative 100% intensity at 0 h.

First, after refreshing the medium, we determined the optimal time for the harvest of the medium. Time-course studies revealed that the secretion of VLDL (apoB) from HepG2 cells into the medium increased linearly up to 40 h (data not shown). Therefore, we performed our analyses by incubating the cells for 24 h. EA was dissolved in dimethyl sulfoxide (DMSO) and added to the serum-free medium, ensuring that the final concentration of DMSO was below 1%. EA suppressed the secretion of VLDL (apoB) at the 50 µM concentration compared to the secretion in the untreated control using ELISA (Figure 3a). This was also confirmed by western blot analysis of the culture medium (Figure 3b).

### 2.3. Effect of EA on the Secretion of apoA-1 from HepG2 Cells

**Figure 4:**
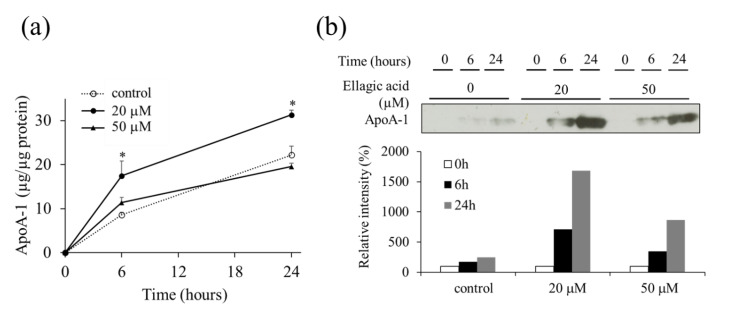
Effect of ellagic acid (EA) on the secretion of apoA-1 from HepG2 cells treated for 24 h. (**a**) Concentration of ApoA-1 secreted from HepG2 cells treated with EA in the culture medium was determined by ELISA as described in “Materials and Methods”. (**b**) Western blot analysis showing the levels of apoA-1 secreted from HepG2 cells treated with EA in the culture medium. Values are expressed as mean ± SE (*n* = 3). * *p* < 0.05 vs. control group (without EA treatment). The data was expressed by comparing band intensities in each concentration of ellagic acid to the concentration of 100% relative intensity at 0 h.

The effect of EA on the secretion of apoA-1 was determined by apoA-1 ELISA developed using an anti-apoA-1 antibody (Figure 4a). The levels of apoA-1 secreted from the HepG2 cells incubated for 24 h with 20 or 50 µM EA in the medium were increased compared to those of the control. The results were corroborated by western blot analysis of the culture medium (Figure 4b).

### 2.4. Effect of EA on apoB Levels in HepG2 Cells

**Figure 5:**
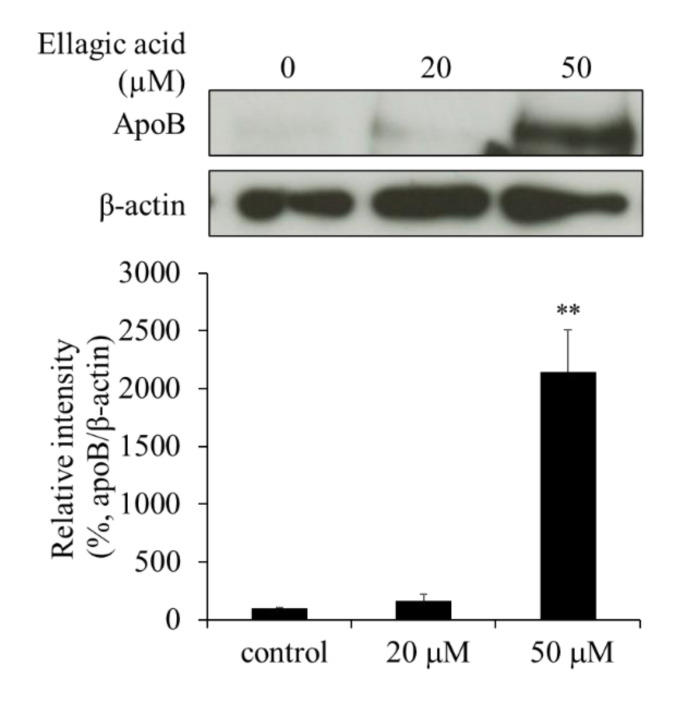
Effect of ellagic acid (EA) on apoB levels in HepG2 cells. Western blot analysis showing apoB levels in HepG2 cells treated with EA. Values are expressed as mean ± SE (*n* = 3). ** *p* < 0.01 vs. control group (without EA treatment).

To clarify how EA suppressed the secretion of apoB from the HepG2 cells, we determined the cellular levels of apoB by western blot analysis. The apoB levels in cells treated with 50 µM EA were significantly higher than those in the control cells (Figure 5). These results indicate that EA suppresses the secretory processes of apoB and that apoB might be retained in the cells.

### 2.5. Effect of EA on apoA-1 Levels in HepG2 Cells

**Figure 6:**
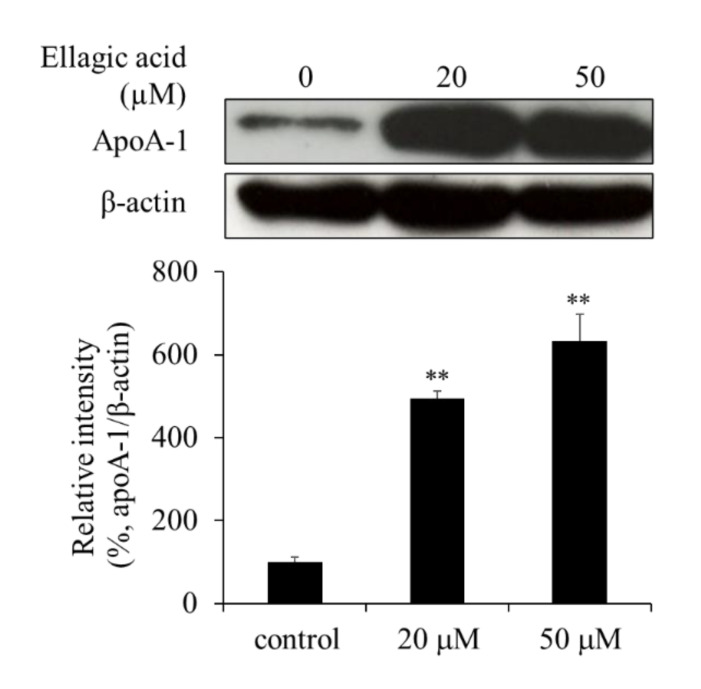
Effect of ellagic acid (EA) on apoA-1 levels in HepG2 cells. Western blot analysis showing apoA-1 levels in HepG2 cells treated with EA. Values are expressed as mean ± SE (*n* = 3). ** *p* < 0.01 vs. control group (without EA treatment).

The levels of apoA-1 in the cells were determined by western blot analysis to assess whether EA affected the expression of EA in HepG2 cells or only its secretion from the cells. The apoA-1 levels in the cells were increased upon EA treatment, which suggests that EA may increase the expression and/or synthesis of apoA-1, resulting in its increased secretion (Figure 6).

### 2.6. Effect of EA on Microsomal Triglyceride Transfer Protein (MTP) Levels

**Figure 7:**
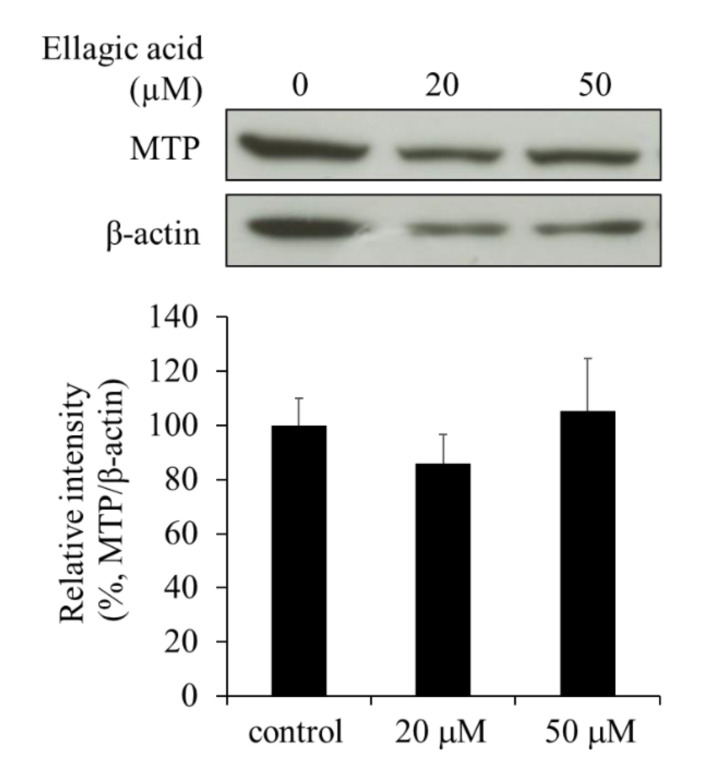
Effect of ellagic acid (EA) on MTP levels in HepG2 cells. Western blot analysis showing MTP levels in HepG2 cells treated with EA. Values are expressed as mean ± SE (*n* = 3). No significant differences were observed between the groups.

In hepatocytes, the process of secretion of VLDLs involves several steps, including lipid synthesis, VLDL maturation, the accumulation of neutral lipids into apoB apolipoproteins, and the secretory traffic. Among these, the transfer of TG to the apoB apolipoprotein is done by MTP, which plays a critical role in the maturation and secretion of VLDLs. Because the secretion of apoB-containing lipoproteins was suppressed by EA, we investigated whether EA also affected the expression of MTP. The expression of MTP in the HepG2 cells was not significantly affected by EA (Figure 7). This suggests that MTP is probably not a major target of EA.

## 3. Discussion

In this study, we investigated the effects of EA on the secretion of two apolipoproteins, apoB and apoA-1, from human HepG2 hepatoma cells. Because apoB is not secreted as such and is instead secreted as VLDL, it can be monitored by measuring the levels of VLDL secreted in the culture medium.

Upon the treatment of HepG2 cells with EA, the amount of apoB secreted in the medium was significantly suppressed compared to that in the control cells. In contrast, the secretion of apoA-1 was significantly enhanced compared to that in the control cells. Moreover, the levels of apoB and apoA-1 in the cells were markedly increased at 24 h of EA treatment. These results suggest that EA inhibits the secretory processes of apoB and that apoB may be retained in the cells. We also showed that EA may promote the expression and/or synthesis of apoA-1 because its levels increased in the medium as well as in the cells. In addition, similar results were obtained using another human hepatoma cell, Huh-7 (data not shown). Therefore, the present observations seem to be a common and reliable event in human hepatocyte cells.

Within hepatocytes, TGs are transferred by MTP and associated with apoB to form VLDLs that are secreted from the liver and transported to peripheral tissues [9]. Due to loss-of-function mutations, the MTP in abetalipoproteinemia does not transfer lipids to VLDLs of small intestinal epithelial chylomicrons and hepatocytes, resulting in profound hypolipidemia [10]. Thus, when the levels of MTP are reduced or when the protein is dysfunctional, it inhibits the release of VLDLs. However, in the present study, EA was found to have no effect on the expression of MTP. Thus, it is unlikely that EA reduces the secretion of VLDLs through the inhibition of MTP. However, the possibility that EA suppresses the function of MTP cannot be excluded.

Recently, several polyphenols have been reported to inhibit the secretion of VLDLs and apoB from human hepatocytes [11,12]. Although the mechanisms are not fully understood, it has been suggested that these polyphenols can act at multiple points. For example, the inhibitory effect of naringenin from grapefruit on the secretion of apoB from HepG2 cells was reported to involve an increase in the expression of the low-density lipoprotein receptor (LDLr) by inhibiting the MTP activity or by upregulating the expression of SREBP-1 [13,14]. Previously, EA (25 μM) was reported to reduce the secretion of VLDLs in the medium by upregulating LDLr and downregulating MTP mRNA levels in HepG2 cells and the secretion of apoB [15]. Although we observed a similar phenomenon in the present study, we did not notice a distinct effect on the intracellular levels of MTP (Figure 7). This may be due, in part, to the differences in the methods used for detection, such as western blotting and RT-PCR. In addition, we observed that apoB was retained in the cells treated with EA. Indeed, EA has been reported to inhibit the fusion of the vesicles and target membranes by being inserted into the soluble *N*-ethylmaleimide-sensitive factor attachment protein receptor (SNARE) complex in vitro [16]. Therefore, EA affects the mechanism for the secretion of apoB (VLDL) and apparently suppresses its secretion. Generally, apoB proteins are rapidly degraded by proteasomes after ubiquitination by the ER protein quality-control systems. However, we found that apoB accumulated in the cells, suggesting that its accumulation possibly occurs in secretory vesicles other than the ER.

Thus, we demonstrated that EA could suppress the secretion of apoB (VLDL) from hepatocytes, although the mechanism remains unclear. A similar phenomenon may also occur in the secretion of chylomicrons from the small intestinal epithelial cells. High intravascular concentrations of VLDLs and chylomicrons are risk factors for atherosclerosis. Therefore, oral intake of EA may cause a decrease in the levels of serum lipids and promote human health. Further in vivo and clinical studies using laboratory animals and humans are needed to confirm this hypothesis.

Interestingly, in the present study, EA influenced not only apoB but also the secretion of apo A-1. Apo A-1 is a major protein component of HDLs. Its levels are known to correlate positively to those of high-density lipoprotein cholesterol (HDL-C) and negatively with atherosclerosis. The cardioprotective effects of HDLs have been largely attributed to the capacity of apoA-1 to remove excessive cholesterol and to retro-transport it to the liver [17,18]. In vascular cells, apoA-1 prevents the oxidative modification of LDLs [19]. Indeed, increasing the expression of the apoA-1 gene in ApoE-deficient mice markedly suppressed atherosclerosis [20]. Linoleic acid, an unsaturated fatty acid, also stimulated the secretion of apoA-1 from human colon cancer-derived Caco-2 cells by activating PPAR-gamma [21]. Herein, we also show that EA could enhance the secretion of apoA-1 from HepG2 cells, and the mRNA levels of hepatic apoA-1 also increased upon the oral administration of EA in in vivo experiments using diabetic mouse models [22]. Thus, these results suggest that EA may help improve cardioprotective effects and lipid metabolism in humans by promoting the expression and secretion of apoA-1.

EA specifically suppressed the secretion of apoB and not apoA-1. Although the cellular secretion mechanisms of individual lipoproteins and apolipoproteins are not well understood, several studies have suggested differences in the pathways for the secretion of apolipoproteins [23,24], and differences in the effects of EA on the secretion of apoA-1 and apoB may also be explained by differences in the pathways for apolipoprotein secretion.

The concentration of ellagic acid used in this study is considerably higher than the expected blood concentration. Therefore, there is a limitation in the results obtained in this study. However, since ellagic acid is a ubiquitous polyphenol that is present in many fruits and vegetables, the physiological effects that were observed in this study might occur by consuming a diet rich in vegetables and fruits and their concentrated beverages and supplements.

Clinical studies in humans using foods such as pomegranate extract containing ellagic acid are extremely important in providing useful information on the actual human diet. Although there are few studies in total, several clinical studies on the oral intake of pomegranate extract for human blood lipid levels and hypertension have been reported, and recent studies reporting their meta-analysis have also been published [25,26,27,28,29,30,31,32]. At this time, these treatises do not show a clear effect of pomegranate extract on human health, but some have reported benefits such as the improvement of certain inflammatory parameters [32]. By refining the selection of subjects, test design, test scale, evaluation items, properties of the pomegranate sample used, etc., it is possible that results showing effectiveness may be obtained in the future. In order to clarify such a practical evaluation of these benefits in humans, the results of in vitro studies using human cells such as the current study may provide a certain benefit.

We conclude that EA suppresses the secretion of apoB from HepG2 cells through a mechanism involving interference with the secretory machinery and enhances the secretion of apoA-1 through a mechanism involving the increased expression of the gene/protein. However, further studies are needed to clarify the detailed molecular mechanisms underlying these effects.

## 4. Materials and Methods

### 4.1. Sample Preparation

Ellagic acid (EA) was purchased in powder form from FUJIFILM Wako Pure Chemical Corporation (Osaka, Japan). The chemical structure of EA is shown in Figure 1. It was first diluted in dimethyl sulfoxide (DMSO) and then added to the culture medium at final concentrations of 20 and 50 µM. The final DMSO concentration was adjusted to 1%.

### 4.2. Cell Culture and Sample Treatment

HepG2 cells were purchased from the American Type Culture Collection (Manassas, VA, USA). The cells were maintained in Dulbecco’s modified Eagle medium (DMEM) (Nissui Pharmaceutical Co., Tokyo, Japan) and supplemented with 10% fetal bovine serum (FBS) (Gibco™, Life Technologies, Frederick, MD, USA), 1% penicillin–streptomycin mixed solution, 200 mM glutamic acid, 100 mM pyruvic acid, 4.5 g/L glucose, and 7.5% sodium carbonate at 37 °C under an atmosphere of 5% CO_2_ and seeded on 6-well plates. At 80% confluency, the cells were washed twice with sterile phosphate-buffered saline (PBS) and incubated with the serum-free medium containing EA at the required concentration for 24 h. Control cells were treated with 0.1% DMSO (final concentration in the medium) as a vehicle.

### 4.3. Cytotoxicity Assay

The cytotoxicity of EA was measured using the CellTiter96 AQ_ueous_ One™ Solution Cell Proliferation Assay kit (Promega Corporation, Fitchburg, WI, USA).

### 4.4. ELISA for Determination of apoB and apoA-1 Levels in the Culture Medium

We developed a sandwich ELISA for the quantification of apoB-containing VLDLs and apoA-1 secreted from HepG2 cells in the culture medium. To this end, a murine monoclonal antibody (Monosan, Uden, The Netherlands) against human VLDL was used as the capture antibody. To detect the antibodies, goat polyclonal anti-human apoB (VLDL) antibody (Rockland, PA, USA) was used. The antibody (Ab)–Antigen (Ag) complex was detected using horse radish peroxidase (HRP)-labeled anti-goat IgG (Promega Corporation). Three washes with 200 μL of PBS-T (PBS with 0.1% [*v/v*] Tween 20) were followed by incubation with 100 μL of tetramethylbenzidine (TMB) peroxidase substrate (Kirkegaad & Perry Laboratories, Gaithersburg, MD, USA) until the development of color, followed by the addition of 100 μL of 1 M phosphate to stop the reaction and signal amplification. The absorbance of the yellowish solution was read at 450 nm using a Multilabel Counter 1420 ARVOsx-1 (PerkinElmer Life Sciences, Waltham, MA, USA). The prepared standard curves were used to determine the concentration of human VLDL in the samples. Purified human VLDL (Chemicon, MA, USA) diluted with the same cell culture medium was used to prepare the standard curves. In this ELISA, human VLDL could be quantified in the 5–1000 ng/well range (data not shown). Similarly, apoA-1 levels were measured using the following reagents: mouse anti-human apolipoprotein A-1 monoclonal antibody (Merck Millipore, MA, USA) was used as a capture antibody, and anti-apolipoprotein A-1 rabbit pAb (Merck Millipore) was used as a detection antibody. Native human apolipoprotein A-1 (Bio-Rad, CA, USA) was used for preparing the standard curve.

### 4.5. Sodium Dodecyl Sulfate Polyacrylamide Gel Electrophoresis (SDS-PAGE) and Western Blot Analysis

Cell lysate was boiled for 5 min in SDS sample buffer [33]. Proteins (30 µg/lane) were separated by SDS-PAGE and transferred onto polyvinylidene difluoride membranes (Immobilon™-P, Merck Millipore). Antibodies against apoB (Chemicon), ApoA-1 (MerckMillipore, MA, USA), HRP-labeled anti-mouse IgG (Promega Corporation), and HRP-labeled anti-rabbit IgG (Thermo Fisher Scientific Inc.) were used for western blotting. All immunoreactive proteins were detected using ECL™ Western blotting Detection Reagents (GE Healthcare, Buckinghamshire, UK).

### 4.6. Statistical Analysis

Statistical differences were determined using the Tukey–Kramer test. Differences were considered significant at *p* < 0.05. Statistical analyses were performed using Stat View v. 5.0 (SAS Institute, Tokyo, Japan).

## Data Availability

The data presented in this study are available upon request from the corresponding author.
